# 

**Published:** 1879-03

**Authors:** 


MARCH, 1879.

### THE AMERICAN JOURNAL OF DENTAL SCIENCE,

S.G.O.L. INDEXED

EDITED BY

F. J. S. GORGAS, M. D., D. D. S.,

AND

JAMES B. HODGKIN, D. D. S.

PUBLISHED MONTHLY.

$2.50 PER ANNUM.

VOL. 12.—THIRD SERIES.—No. 11.

BALTIMORE:
SNOWDEN & COWMAN.

LONDON:

TRUBNER & CO., 60 PATERNOSTER ROW.

1879.

PAYABLE IN ADVANCE.

